# Effectiveness of a Brief Digitized Contact-Based Intervention in Improving Mental Health Stigma and Help-Seeking in Young Adults: Mixed Methods Study

**DOI:** 10.2196/74391

**Published:** 2026-06-10

**Authors:** Annabel Songco, Natalie Dahora, Andrew Essen, Andrew Mackinnon, Gemma Sicouri, Elizabeth Pellicano, Jennifer L Hudson

**Affiliations:** 1 Black Dog Institute, UNSW Sydney, Hospital Road, Randwick, Sydney, New South Wales, 2031, Australia, 61 0290659251; 2 Department of Psychology, Macquarie University, Sydney, New South Wales, Australia; 3 Department of Clinical, Educational, and Health Psychology, University College London, London, United Kingdom

**Keywords:** contact-based intervention, mental health, stigma, help-seeking, young adults, lived experience

## Abstract

**Background:**

Contact-based interventions, where individuals share their lived experiences of mental health difficulties and recovery, appear effective in reducing stigma.

**Objective:**

This study examined the effectiveness of a brief digitized contact-based intervention in reducing mental health stigma and improving help-seeking intentions in young adults. The study also sought a deeper understanding of the perspectives of young adults toward the contact-based intervention.

**Methods:**

A mixed methods study, using quantitative and qualitative analyses, examined mental health public stigma, self-stigma, and help-seeking attitudes in undergraduate psychology students (N=328) before, immediately after, and 1 week following the contact-based intervention, compared to an active control. The intervention comprised a brief video (8 min) of a mental health consumer sharing their lived experience of mental health difficulties and their recovery. Participants were invited to participate in a subsequent semistructured interview (n=12) that further explored their perspectives on the contact-based intervention.

**Results:**

Relative to control participants, those in the intervention group reported small improvements in mental health public stigma and help-seeking intentions using formal sources of help; however, this was not maintained at 1-week follow-up. There was limited evidence that the intervention improved self-stigma or help-seeking intentions using informal sources of help. Improvements in self-stigma were not evident following the intervention, but were observed 1 week later. Additional exploratory analyses showed that participants with high mental distress were more likely to provide negative evaluations of the intervention in terms of the helpfulness of the videos, relatability to the speaker, and showed a decrease in their desire to seek help from friends and family following the videos, compared to those with low mental health distress.

**Conclusions:**

Although this brief digitized contact-based intervention holds promise as an easily disseminated strategy for young adults to reduce mental health public stigma and improve formal help-seeking intentions, these effects show that overall improvements in public stigma and formal help-seeking intentions were short-lived. Furthermore, the potential negative impact of the intervention on informal help-seeking intentions in those with high mental health distress suggests a need to tailor interventions for young adults with high levels of anxiety or depression. The study has important implications for designing brief contact-based interventions and what young adults would benefit from the most over time.

## Introduction

### Background

Mental health stigma is a significant deterrent to individuals with mental disorders from seeking help through health services [1]. Both public stigma (negative attitudes and stereotypes toward others or groups) and self-stigma (internalized negative attitudes) have a negative impact on help-seeking behaviors and appear to disproportionately impact youth [2]. Stigma is a key barrier to help-seeking behavior, indirectly contributing to the maintenance of untreated mental health difficulties and associated economic and social costs.

Contact-based interventions are one of the most effective strategies for improving mental health stigma [3,4]. Contact-based interventions involve either direct or indirect interaction with an individual from the respective marginalized group to allow opportunity for the disconfirmation of stereotypes and subsequent reduction of prejudicial attitudes [5]. These interventions were derived from Allport’s [6] theory of intergroup contact, based on the premise that interaction of a positive nature between individuals of majority and minority groups works to reduce prejudices. The aim of these interventions is to reduce prejudicial beliefs and attitudes through interpersonal contact, which typically involves an individual with lived experience of mental health difficulties sharing and discussing their personal experience. Contact-based interventions can involve varying degrees of interpersonal engagement and activate different psychological mechanisms for attitude change. When evaluating randomized controlled trials, Corrigan and colleagues [5] found that contact-based interventions delivered a small-to-moderate reduction in stigma and were better than education interventions for reducing stigma in adults. Further meta-analyses found pure contact-based interventions to be comparable in their effectiveness with combined contact and education interventions [7], although there is some question about the long-term maintenance of effects [8].

The effectiveness of contact-based interventions does not appear to differ across modalities, such as face-to-face or video. With the increasing accessibility of technology, the growing popularity of telehealth, online services, and immersive technologies, such as virtual reality in the psychological and medical fields, there is a rising interest in the efficacy of scalable, easily implementable digital stigma reduction interventions that are capable of reaching broad audiences. Several studies have found that when compared to live contact-based interventions, interventions consisting of video interpersonal contact have proven to be just as efficacious [7,8]. A review by Janoušková and colleagues [9] showed that video interventions for young adults, which included social contact, were comparable to or better than traditional modalities for improving stigma. However, most studies focused on evaluating the effectiveness of contact-based interventions in addressing public stigma, with few studies on self-stigma, highlighting a gap in the literature [10]. In a young adult sample, one study of a prolonged intervention comprising five 2-hour workshops found that contact significantly improved self-stigma [11]. However, little is known about the effectiveness of brief, more easily disseminated interventions on self-stigma for this population. Given the role of both public and self-stigma in mitigating help-seeking behavior, these factors are crucial to appropriately evaluate and develop antistigma programs, with further research needed on the optimal duration of the intervention and effectiveness of brief interventions.

Few studies have investigated improvements to help-seeking following contact, beyond stigma improvement [12]. Encouragingly, recent studies targeting young adults have found improvements in help-seeking intentions following pure video contact and combined contact and education interventions [13,14]. Factors such as openness to seeking help and increased mental health literacy have improved help-seeking intentions and attitudes in young adults. Moreover, mental health stigma research has predominantly focused on stigma toward more severe psychiatric illnesses. For example, individuals with schizophrenia are often stigmatized and perceived as dangerous, unstable, or unpredictable, with lower likelihood of recovering from their disorder [15]. Research focused on more common mental health difficulties, such as anxiety and depression, is limited, despite being the leading cause of mental health burden in young people [16]. In a recent review, Curcio and Corboy [17] indicated that individuals with mood or anxiety disorders are perceived differently, with stigma relating to perceptions around fault and responsibility [18]. Thus, the nature and intensity of mental health stigma can differ across mental disorders and further research on contact-based interventions that target common mental health difficulties, such as anxiety and depression, is needed.

A further critical gap in the literature concerns the evaluation of moderating variables that may enhance the effectiveness of interventions. Some studies have found participant variables such as age, gender, and ethnicity to moderate the effectiveness of contact interventions [19]. Contact theory stipulates that similarity and equality between the individual of the stigmatized group and the majority group member maximizes the applicability and credibility of the intervention and resulting efficacy [20]. Indeed, Kruger and colleagues [13] found that tailoring contact intervention videos to college students by using individuals who were college students themselves increased help-seeking intentions, while the generic contact intervention did not.

Another factor to consider when tailoring contact-based interventions is an individual’s previous or current experiences with mental health difficulties, which can impact subsequent help-seeking behaviors. Brennan and McGrew [21] found differences in how mental health consumers and nonconsumers perceived a contact-based intervention, with nonconsumers significantly more likely to find the intervention educational, endorse feelings of encouragement, and express beliefs that recovery is possible. Further, Clement et al [1] reported that when discussing mental health stigma, individuals with previous mental health difficulties were more likely to report feelings of shame and embarrassment and less likely to report weakness or social rejection, compared to those without previous mental health difficulties. Cerully and colleagues [22] reported that while contact-based interventions are effective for those with and without personal experiences of mental health difficulties, they are less effective for those with prior personal experiences. Thus, there is currently a gap in the literature with few studies investigating how individual characteristics, such as previous or current mental health difficulties, may impact the efficacy of the intervention.

### This Study

The batyr@uni program, run by the mental health organization batyr, is a contact-based intervention aimed at reducing general mental health stigma and increasing help-seeking for university students. The intervention is delivered to university students as well as to schools, with a focus on common mental health difficulties such as anxiety and depression. The batyr@uni program (60‐90 min in duration) can be delivered face-to-face or online and focuses on sharing the lived experience stories of individuals with mental health difficulties and their subsequent recovery via help-seeking. The lived experience stories (delivered live or through brief digital videos) provide valuable insights into how young adults can support their peers, strategies, and self-care practices to maintain good mental health, help identify signs and symptoms of mental ill health, and access support. The program involves young people from diverse backgrounds (eg, age, gender, and geographical context) and experiences to optimize relatability to audience members. During the COVID-19 pandemic, the batyr@uni program transitioned from traditional face-to-face to online video delivery.

The aim of this study was to quantitatively examine the effectiveness of one component of the digitized contact-based intervention (ie, an 8-min video of lived experience stories targeting anxiety and depression) in reducing mental health stigma and improving help-seeking intentions in young adults. We also used a qualitative approach to provide a deeper understanding of young adults’ perspectives on the brief contact-based intervention. Further, the study aimed to identify potential differences in whether young adults experiencing high or low mental health distress respond differently to the intervention.

Based on previous literature, it was hypothesized that those in the intervention group would experience greater reduction in both public and self-stigma, and greater improvements in help-seeking intentions (using formal and informal sources of help), relative to the active control group, and that these effects would be maintained at 1-week follow-up. In addition, it was hypothesized that individuals experiencing high mental health distress would perceive mental health stigma differently and find the intervention more beneficial, compared to individuals with low mental health distress. For the qualitative data, we used reflexive thematic analysis to identify themes around participants’ perceptions of the intervention [23].

## Methods

### Participants

Participants were undergraduate psychology students (N=328) aged 17 to 24 (mean 18.95, SD 1.61) years. The majority of participants were female (269/328, 82%) and self-identified their ethnicity as Caucasian (173/328, 52.7%), Asian (77/328, 23.5%), self-identified (35/328, 10.7%), Middle Eastern (31/328, 9.5%), African (6/328, 1.8%), Hispanic (5/328, 1.5%), and Aboriginal and/or Torres Strait Islander (5/328, 1.5%). From this larger sample, 12 participants volunteered to participate in a subsequent qualitative study. These participants were aged 18 to 24 (mean 20.75, SD 2.70) years, and 5 identified as male, 5 as female, and 2 reported their gender as “Self-identified.” Half of the participants identified as Caucasian (n=6) and half as Asian (n=6). Participants were recruited via a university research portal and received course credit in return for their participation. The study was advertised to first-year psychology undergraduate students through the university research portal and was conducted online via the LimeSurvey platform (LimeSurvey GmbH). A G-Power analysis was used to determine adequate sample size (power=.95, Cohen*d*=0.25, and α=.05).

### Study Design

**Figure 1.**
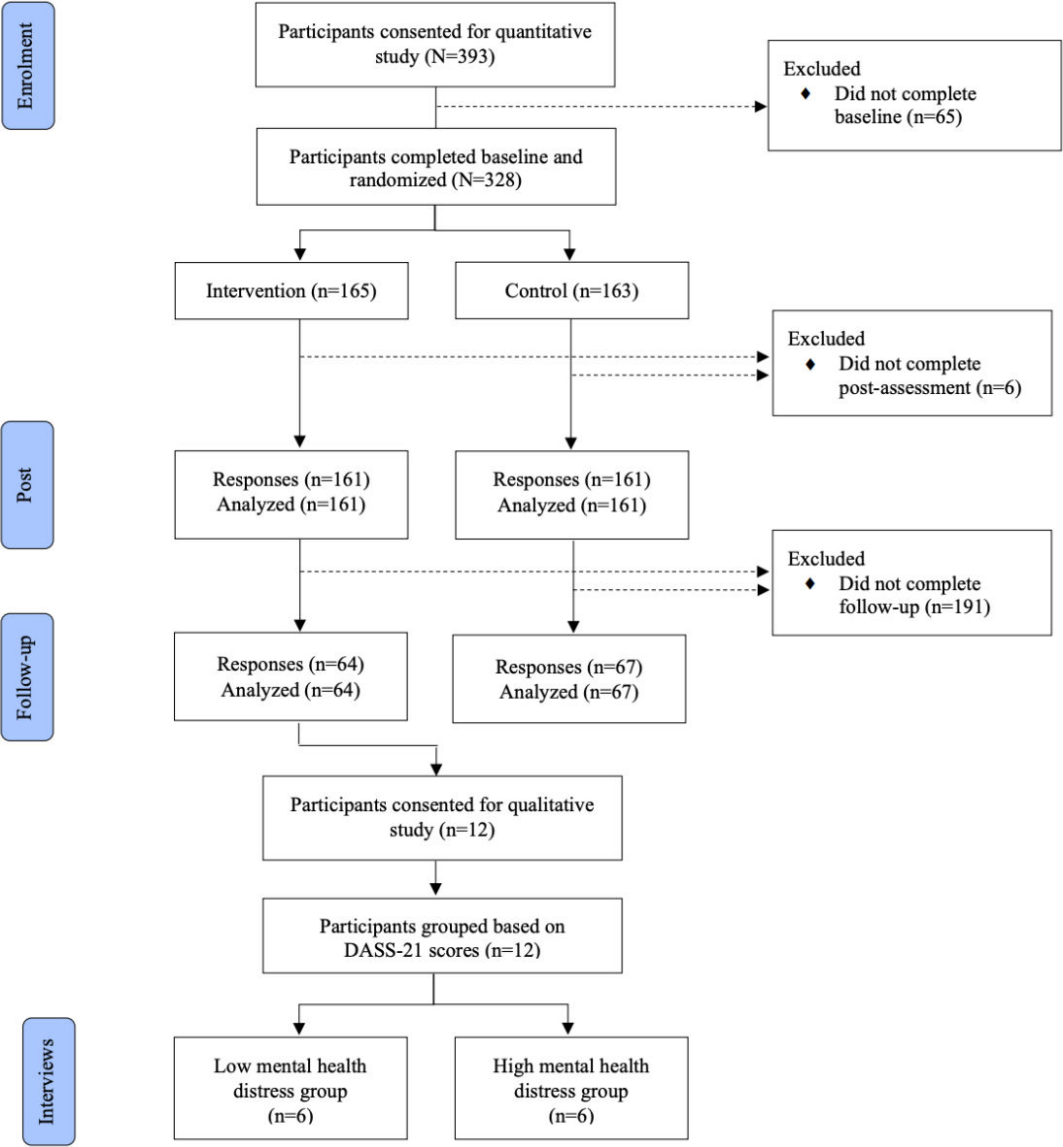
Study flow (reason for withdrawals and noncompleters unknown due to the anonymous nature of the online study). DASS-21: Depression, Anxiety, and Stress Scale - Short Form.

Quantitative data were collected at 3 time points (baseline, postintervention, and 1-wk follow-up) to evaluate the effectiveness of a brief digitized contact-based intervention (one component of the batyr@uni program) in reducing mental health stigma and improving help-seeking intentions in young adults. In addition, participants were invited to participate in a subsequent qualitative study comprising a semistructured interview relating to their perspectives and attitudes about the contact-based intervention. This mixed methods study used both a quantitative and qualitative approach to provide a deeper insight into mental health stigma in young adults (refer to Figure 1 for study flow).

### Ethical Considerations

This study was approved by the Macquarie University Human Research Ethics Committee (reference no. 52021904124875). As the authors changed institutions after data collection, but before analysis could be completed, the study was also reviewed and approved by the University of New South Wales Research Ethics and Compliance Committee (reference no. iRECS7757). This study was not initially defined as a clinical trial by the Macquarie University Human Research Ethics Committee as participants were randomly allocated to receive 2 educational videos designed to change attitudes rather than biomedical or health outcomes. As a result, the study was not preregistered as a clinical trial. However, following the University of New South Wales’ broader definition of a clinical trial, the study was retrospectively registered with the International Standard Randomized Controlled Trial Registry (reference no. ISRCTN65081246). Informed consent was obtained from all participants prior to participation, with the right to withdraw at any time. Participant privacy and confidentiality were maintained through deidentification of data, and participants received course credit in return for their participation.

### Quantitative Study

#### Intervention

Participants allocated to the intervention group viewed 1 of 4 videos (8 min) of a mental health consumer sharing their lived experience of mental health difficulties, specifically relating to anxiety and depression, and their recovery. The videos provided by batyr are being delivered as part of their mental health stigma intervention, the batyr@uni program, and included a short prerecorded debrief by a batyr representative who emphasized key themes. To increase diversity of the speakers across the control and the intervention group, 4 versions of the video were used, varying the gender and perceived racial appearance of the speaker: a female speaker with a White racial appearance, a male speaker with a White racial appearance, a female speaker with a non-White racial appearance, and a male speaker with a non-White racial appearance. Racial characteristics were coded based on visual appearance only; speakers did not self‑report race or ethnicity. All speakers were young adults, approximately matching the age range of the study population. Participants were randomly allocated to watch 1 of the 4 videos.

#### Active Control

Participants in the control group viewed 1 video (8 min) of an individual speaking about the benefits of healthy eating. The information on healthy eating was adapted from an Australian Government resource (Australian National Health and Medical Research Council, 2013), and all videos followed the same script. The individual speaker in the video for the active control group was matched with the video in the intervention group in terms of approximate age, gender, and ethnicity.

### Qualitative Study

Participants were eligible to participate in the qualitative study if they had previously completed the quantitative study and were allocated to the intervention group. Participants in the intervention group were provided with an opportunity to express their interest in participating in an interview to discuss their perspectives on the brief contact-based intervention.

### Measures

#### Demographics

Demographics such as age, gender, ethnicity, and whether participants identified as Aboriginal or Torres Strait Islander are reported in Table 1.

**Table 1.** Demographic characteristics of participants at baseline.

Variable	Intervention	Control	Total
Age, mean (SD; range)	18.91 (1.51; 17-24)	18.96 (1.67; 17-24)	18.95 (1.61; 17-24)
Sex, n (%)			
Female	143 (43.6)	126 (38.4)	269 (82)
Male	19 (5.8)	33 (10.1)	52 (15.9)
Do not want to say	3 (0.9)	0 (0)	3 (0.9)
Self-identified	0 (0)	4 (1.2)	4 (1.2)
Ethnicity, n (%)			
Caucasian	92 (28)	81 (24.7)	173 (52.7)
Asian	35 (10.7)	42 (12.8)	77 (23.5)
Self-identified	18 (5.5)	17 (5.2)	35 (10.7)
Middle Eastern	14 (4.3)	17 (5.2)	31 (9.5)
African	3 (0.9)	3 (0.9)	6 (1.8)
Hispanic	2 (0.6)	3 (0.9)	5 (1.5)
Don’t want to say	1 (0.3)	0 (0)	1 (0.3)
Aboriginal and/or Torres Strait Islander, n (%)			
No	162 (49.4)	160 (48.8)	322 (98.2)
Yes	3 (0.9)	2 (0.6)	5 (1.5)
Don’t want to say	0 (0)	1 (0.3)	1 (0.3)

#### Public Stigma

Mental health stigma was measured with a modified version of the Generalized Anxiety Stigma Scale (GASS)—Personal Stigma Subscale. This 10-item scale measures public stigma related to anxiety [24] and was adapted in this study to measure general mental health stigma. To achieve this, the word “anxiety” in each item was replaced with “mental health problems” to create a new Mental Health Stigma Scale (MHSS).

Participants rated each item on a 5-point scale ranging from strongly disagree to strongly agree. The total score is the sum of all items, with higher scores indicative of greater stigma toward mental health. This measure has demonstrated good internal consistency, test-retest reliability, and validity in adults [24,25] and young adults [26]. In this study, the modified MHSS showed good reliability across time points (Cronbach α=0.84, 0.89, and 0.89) and high correlations with the GASS at each time point (ranging from *r*=0.80 to 0.90).

#### Self-Stigma

The Self-Stigma of Mental Illness Scale (SSOMI) measured internalized stigma of mental illness [27]. Participants rated 10 items on a 5-point scale ranging from strongly disagree to strongly agree. Items are summed to derive a total score, with higher scores indicative of greater self-stigma of mental illness. As the SSOMI uses future-tense language (ie, “I would feel...”), the questionnaire could be used with individuals who do not currently have clinical levels of a mental health diagnosis, and as a result, this is best considered as anticipated self-stigma. The SSOMI has good internal consistency, test-retest reliability, and validity in young adults [27,28]. The SSOMI in this study showed good reliability across time points (Cronbach α=0.81, 0.82, and 0.87).

#### Help-Seeking Intentions

The General Help-Seeking Questionnaire (GHSQ) assesses intentions to seek help [29]. Participants were asked, “if you were having a personal or emotional problem, how likely is it that you would seek help from each of the following sources?” Participants indicated how likely, on a 7-point scale (extremely unlikely to extremely likely)*,* they would seek help from each source. The GHSQ contains two subscales: (1) mean intentions to seek formal sources of help (ie, mental health professional, phone helpline, family doctor or general practitioner [GP], and teacher or tutor) and (2) mean intentions to seek informal sources of help (ie, partner, friend, parent, relative, or family member). Higher scores indicated greater help-seeking intentions. The GHSQ has demonstrated good internal consistency, test-retest reliability, and validity in young adults [30]. In this study, the formal subscale yielded good reliability (Cronbach α=0.80, 0.81, and 0.80), while the informal subscale demonstrated low reliability (Cronbach α=0.51, 0.55, and 0.55).

#### Mental Health Distress

The Depression, Anxiety, and Stress Scale - Short Form (DASS-21) is a 21-item self-report measure that assesses depressive, anxious, and stress-related symptoms [31]. Participants rated each item on a 4-point scale (ie, never, sometimes, often, and almost always), indicating the extent to which each item applied to them over the past week. Total scores were summed into 3 subscales (depression, anxiety, and stress), with higher scores indicating greater distress; categorized into normal, mild, moderate, severe, and extremely severe based on the DASS manual. The DASS-21 has consistently demonstrated good reliability [25,32] and excellent internal reliability in this study (Cronbach α=0.95).

### Procedure

Prior to the study, participants were provided with participant information and consent forms that included details about the quantitative study. Once informed consent was obtained, participants completed demographic questions (age, gender, and ethnicity) and baseline assessments (public stigma, self-stigma, help-seeking intentions, and current levels of mental health distress). Participants were then randomly allocated to an intervention group (ie, brief digitized contact-based intervention video) or an active control group (ie, healthy eating video) through random number generation implemented in the LimeSurvey platform. After viewing the full video, participants completed assessments immediately after the video and at 1-week follow-up.

After completing the quantitative study, participants had an opportunity to participate in a qualitative study involving an interview relating to their perspectives and attitudes about the intervention. Participants completed individual semistructured interviews with a researcher through Zoom (Zoom Communications, Inc). Each participant was asked the same open-ended questions relating to their previous relevant experiences, help-seeking attitudes, perspectives on the contact-based intervention, and thoughts about the speaker in the intervention (refer to Multimedia Appendix 1). Participants were asked additional follow-up questions to provide further clarification. All Zoom interviews (26 min on average, ranging from 17 to 32 min) were audio-recorded with the participants’ permission and transcribed verbatim.

### Data Analysis

#### Quantitative Study

This study used mixed model analysis of covariance (ANCOVA), with time (baseline, postintervention, and 1-wk follow-up) as the within-subjects factor and group (intervention or active control) as the between-subjects factor.

Despite random assignment after the completion of baseline measures and no differences in the way individuals progressed through the trial, there were substantial baseline differences between groups on outcome measures. Accordingly, to examine the effect of the intervention on the outcome measures (MHSS, SSOMI, and GHSQ), ANCOVA using baseline status on the outcome variable as a covariate was chosen as the most appropriate method to adjust for baseline imbalance. Baseline values of each outcome were centered at the whole-sample (intention-to-treat) mean at baseline, so the model parameters for group represent the effect of the intervention at the group mean. The effect of the covariate was allowed to differ postintervention and at follow-up, as was the effect of the intervention itself. Standardized effect sizes were calculated using the difference between groups at the mean and dividing it by the modeled pooled SD for the variable at each time point. Effect sizes (the strengths of the effects demonstrated) were calculated using Cohen *d* estimates of 0.2 for small effect sizes, 0.5 for medium, and 0.8 for large [33].

These analyses were repeated separately for participants with baseline total scores on the DASS-21 of 14 or higher (ie, high mental health distress group) and those with scores below 14 (ie, low mental health distress group) to explore the effectiveness of the intervention in these subgroups.

#### Qualitative Study

Participants (n=12) were divided into 2 groups based on their levels of current distress to identify potential qualitative differences between individuals with high mental health distress (total scores>14 on the DASS-21) and low mental health distress (total scores<14 on the DASS-21). Groups consisted of an equal number of participants (n=6). An explanatory sequential design was used to integrate the quantitative and qualitative findings. Quantitative data were collected and analyzed first, followed by qualitative interviews with a subset of participants who volunteered to elaborate on their experiences with the intervention. This approach allowed for a deeper understanding of unexpected or complex results by examining whether participants with high mental health distress responded differently to the intervention compared to those with low mental health distress.

We followed Braun and Clarke’s [23] method of reflexive thematic analysis within a contextualist framework, which emphasizes that a person’s meaning-making occurs through an interplay between the individual and their environment. In so doing, we used an inductive (bottom-up) approach to identify patterned meanings within the dataset.

One lead researcher conducted all qualitative interviews and transcriptions, immersing themselves in the data. Once completed, participant transcripts were separated into 2 groups (“low mental health distress” or “high mental health distress”) and read and reread, with reflexive notes (memos) made relating to notable or recurring observations or concepts. Codes were then applied to each individual transcript, line by line. These codes were discussed during regular meetings with another member of the research team, at which points some codes were modified and reapplied to the transcripts. Given the significant overlap in codes and potential themes, the groups (low and high mental health distress) were combined for subsequent analysis. To identify candidate themes and subthemes, the lead researcher grouped codes together, focusing on semantic features of the data. These initial themes and subthemes were further refined during regular discussions with the broader team, and eventually drawn together into a thematic map. Relevant data, including direct quotes from the transcripts, were collated under the respective themes and subthemes to demonstrate theme or subtheme coherence. The thematic map and the quotes supporting it were reviewed several times with the broader team prior to writing up the results. Consistent with reflexive thematic analysis methodology, we did not focus on data saturation, as we recognize that decisions about sample size and the number of data items collected are inherently subjective and context-dependent, and cannot be fully determined before analysis [23]. Instead, our sampling approach was guided by the concept of “information power” [34] in qualitative research, which considers sample size based on the study aim, sample specificity, theory, data richness, and relevance.

## Results

### Quantitative Findings

#### Primary Analyses

##### Public Stigma

There was a significant difference in mental health public stigma of 0.76 points between the intervention and the active control group postintervention (*F*_1, 319_=8.53, 95% CI 0.25‐1.27; *P*=.004). This represented a small effect size (0.18) in favor of the intervention.

##### Self-Stigma

There was no significant difference between the intervention and active control groups on self-stigma postintervention (modeled mean difference=0.37, SD 6.88; *F*_1, 319_=0.74, 95% CI –0.47‐1.22; *P*=.39). However, there was a significant group difference on self-stigma at 1-week follow-up (modeled mean difference=1.55, SD 6.92; *F*_1, 166.41_=5.76, 95% CI 0.27‐2.82; *P*=.02, showing a small, beneficial effect size (0.22).

##### Help-Seeking Intentions: Formal

There was a significant difference of 0.23 points (SD 1.58) between the intervention and the active control group on intentions to seek help from formal sources of help postintervention (*F*_1, 319_=7.78, 95% CI 0.07‐0.39; *P*=.006), showing a small effect size (0.15) in favor of the intervention. There was no significant group difference in intentions to seek help from formal sources from baseline to 1-week follow-up (modeled mean difference=0.24, SD 1.53*; F*_1, 145.23_=2.13, 95% CI –0.08 to 0.57; *P*=.15).

##### Help-Seeking Intentions: Informal

There were no significant group differences on intentions to seek help from informal sources postintervention (modeled mean difference=–0.07, SD 1.15; *F*_1, 319_=1.72, 95% CI –0.17 to 0.03; *P*=.19) or at 1-week follow-up (modeled mean difference=–0.20, SD 1.20; *F*_1, 139.40_=2.69, 95% CI –0.44 to 0.04; *P*=.10).

Refer to Table 2 for means and SDs of outcome measures across time points.

**Table 2.** Group means and SDs of outcomes for the overall sample and subgroups.

	Baseline		Postintervention		Follow-up	
	n^a^	Mean (SD)	n	Mean (SD)	n	Mean (SD)
Intervention group						
Overall^b^						
Public stigma	165	2.94 (3.57)	161	2.35 (3.36)	64	2.64 (3.57)
Self-stigma	165	33.96 (6.48)	161	33.16 (6.68)	64	32.16 (7.80)
Formal help-seeking	165	3.88 (1.47)	161	3.98 (1.62)	64	3.70 (1.43)
Informal help-seeking	165	4.45 (1.14)	161	4.47 (1.19)	64	4.14 (1.23)
Subgroups^c^,^d^						
Public stigma^e^						
High distress	121	2.88 (3.36)	118	2.14 (3.00)	49	2.53 (3.42)
Low distress	44	3.11 (4.15)	43	2.95 (4.19)	15	3.00 (4.12)
Self-stigma^f^						
High distress	121	34.78 (5.93)	118	33.81 (6.62)	49	34.04 (6.90)
Low distress	44	31.73 (7.44)	43	31.35 (6.59)	15	26.00 (7.59)
Formal help-seeking^g^						
High distress	121	3.83 (1.45)	118	3.90 (1.59)	49	3.57 (1.38)
Low distress	44	4.00 (1.54)	43	4.23 (1.68)	49	4.13 (1.57)
Informal help-seeking^h^						
High distress	121	4.40 (1.09)	118	4.37 (1.15)	49	3.88 (1.19)
Low distress	44	4.59 (1.28)	43	4.77 (1.26)	15	4.98 (0.97)
Control group						
Overall^b^						
Public stigma	163	3.99 (4.52)	161	4.00 (4.88)	67	4.31 (4.70)
Self-stigma	163	33.22 (6.64)	161	32.90 (7.09)	67	33.21 (6.66)
Formal help-seeking	163	3.81 (1.50)	161	3.67 (1.55)	67	3.35 (1.57)
Informal help-seeking	163	4.50 (1.12)	161	4.57 (1.11)	67	4.58 (1.11)
Subgroups^c^,^d^						
Public stigma						
High distress	115	3.86 (4.46)	114	3.95 (4.86)	48	4.35 (4.57)
Low distress	48	4.31 (4.71)	47	4.13 (5.00)	19	4.21 (5.16)
Self-stigma						
High distress	115	34.38 (6.71)	114	33.85 (7.22)	48	34.06 (7.05)
Low distress	48	30.44 (5.61)	47	30.60 (6.27)	19	31.05 (5.09)
Formal help-seeking						
High distress	115	3.73 (1.51)	114	3.62 (1.58)	48	3.33 (1.58)
Low distress	48	3.99 (1.48)	47	3.79 (1.48)	19	3.39 (1.60)
Informal help-seeking						
High distress	115	4.37 (1.13)	114	4.47 (1.12)	48	4.55 (1.10)
Low distress	48	4.80 (1.07)	47	4.84 (1.06)	19	4.67 (1.16)

a n: number of observations.

b Overall: overall sample.

c High distress: high mental health distress group (>14 on the Depression, Anxiety, and Stress Scale - DASS-21).

d Low distress: low mental health distress group (<14 on the Depression, Anxiety, and Stress Scale - DASS-21).

e Public stigma: Mental Health Public Stigma Scale (MHSS).

f Self-stigma: Self-stigma of Mental Illness Scale (SSOMI).

g Formal help-seeking: General Help-Seeking Questionnaire – formal subscale (GHSQ-formal).

h Informal help-seeking: General Help-Seeking Questionnaire – informal subscale (GHSQ-informal).

##### Exploratory Analyses

###### Mental Health Distress

**Figure 2.**
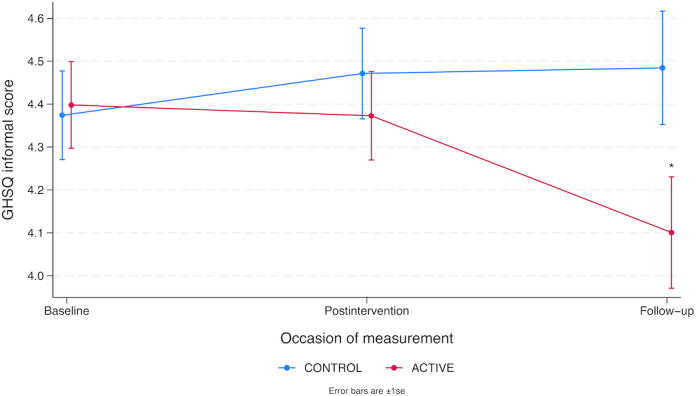
Effect of a significant decrease in informal help-seeking at follow-up for individuals with high mental health distress in the intervention group versus the active control group (*P=.007).

Exploratory subgroup post hoc analyses examined the effectiveness of the intervention for individuals with high mental health distress and low mental health distress (Table 2). Those with high mental health distress showed reduced levels of mental health public stigma (modeled mean difference=1.00, SD 4.01; *F*_1, 229_=11.70, 95% CI 0.43‐1.58; *P*<.001) immediately following the intervention and were less likely to seek help using informal sources of help (eg, a partner, friend, parent, relative, or family member) at 1-week follow-up (modeled mean difference=0.40, SD 1.16; *F*_1, 101.7_=7.56, 95% CI 0.11‐0.69; *P*=.007). This effect of a decrease in intentions to seek help from informal sources, for individuals with high mental health distress was present at follow-up (*P*=.007), but not immediately following the intervention (*P*=.06; Figure 2). The effect of informal help-seeking was not significant in participants with low mental health distress postintervention (*P=.*41) and at 1-week follow-up (*P*=.07). Instead, participants with low mental health distress reported being more likely to seek help from formal sources of help (eg, a mental health professional, phone helpline, GP, or teacher) following the intervention (modeled mean difference=0.41, SD 1.57; *F*_1, 87_=4.41, 95% CI 0.02‐0.79; *P*=.04), but this was not significant at follow-up (*P*=.09).

For individuals with high mental health distress, there was no significant effect of intentions to seek help from formal sources of help following the intervention (*P*=.06) or at 1-week follow-up (*P*=.54). Furthermore, there were no improvements in self-stigma for participants with high mental health distress or low mental health distress, immediately after the intervention (*P=.*39*; P=.*85) or at 1-week follow-up (*P*=.11; *P*=.18), respectively.

### Qualitative Findings

#### Participant Perceptions of the Contact-Based Intervention

We identified two themes regarding participants’ perceptions of the brief digitized contact-based intervention: (1) helpfulness of the intervention and (2) relatability to the speaker (Table 3).

**Table 3.** Themes and subthemes for participant perspectives of the contact-based intervention.

Theme	Subtheme
1. Helpfulness of the intervention	1.1. Helpful for the participant 1.2. Helpful for other people 1.3. Learning something new from the intervention
2. Relatability to the speaker	2.1. Understanding how the speaker would be feeling 2.2. Descriptions of the speaker

#### Theme 1: Helpfulness of the Intervention

Participant responses varied, with both groups indicating mixed feedback on the helpfulness of the intervention. Participants in the high mental health distress group reported that the intervention was not particularly helpful for themselves (subtheme 1.1), with all participants in that group indicating that they did not learn anything new from the intervention (subtheme 1.3). One participant stated that “the video didn’t have a huge impact” and that they “wouldn’t say it was helpful” as they have “seen so many videos like that before” (subtheme 1.1). Another participant stated that the video did not increase their awareness, but served to reinforce “how cruel other people can be toward others because they’re different” (subtheme 1.3). Notably, this lack of perceived benefit from the video was shared by participants in the low mental health distress group with a history of mental health treatment, who voiced similar responses that the video was not helpful “for someone like me who does know quite a bit about it,” and that there was nothing in the video that “I wasn’t aware of” (subtheme 1.3). Conversely, those in the low mental health distress group without a history of mental health treatment generally reported that the video was “definitely helpful,” “enlightening,” or “educational and inspiring” (subtheme 1.1).

While participants in the high mental health distress group reported finding the intervention not helpful for themselves, many indicated that they believed it would be helpful for other people (subtheme 1.2) without their own experiences of mental health difficulties. One participant in the high mental health distress group reported that “for some people, it could be quite helpful, somebody could see it and may be motivated to reach out to someone” (subtheme 1.2). This view was shared by other participants in the same group, who reported that the video might help other people “find and seek some help” (subtheme 1.2). Relatedly, participants in the low mental health distress group with a history of mental health treatment reported similar thoughts that the video might be more helpful for people who “don’t know much about mental health” (subtheme 1.2) or people with higher levels of stigma.

#### Theme 2: Relatability to the Speaker

Participants also spoke at length about the video speaker and the relatability they felt to either the speaker or their story. Importantly, participants in the high mental health distress group reported finding the speaker and their experience less relatable, although they understood how the speaker would be feeling (subtheme 2.1). Several participants in the high mental health distress group stated that while they did not relate to the speaker’s experience or situation, they could relate to the general feeling of having mental health difficulties. For example, one participant stated that they “related to the speaker not in the situation he was in, but in the whole idea of how other people exclude you,” while another noted that they could relate “not in terms of their circumstance, but specifically to how they were feeling” (subtheme 2.1).

Consistent with this view, one participant from the low mental health distress group with a history of mental health treatment reported a similar observation, noting that while they “felt pretty different to the speaker,” they thought “they would probably understand what it feels like to be a little bit different” (subtheme 2.1). Conversely, other participants in the low mental health distress group reported greater relatability to the speaker, with many stating that they could understand the speaker’s situation and relate it to something similar they had previously experienced. Extending on this, one participant in the low mental health distress group stated that they “definitely did relate” and felt “connected” to the speaker, while another participant reported feeling “very empathetic” toward what the speaker was going through (subtheme 2.1).

Furthermore, notable differences arose between groups in descriptions of the speaker (subtheme 2.2). Participants in the low mental health distress group reported feeling strongly about the speaker, often describing them as “real,” “sincere,” “genuine,” “brave,” “heartfelt,” and “engaging” (subtheme 2.2). Participants in the high mental health distress group referred to the speaker as “interesting,” “straightforward,” or “easy to understand” (subtheme 2.2).

## Discussion

### Principal Findings

This study aimed to investigate the effectiveness of a brief digitized contact-based intervention, specifically targeting anxiety and depression, in reducing self-stigma and public stigma and improving help-seeking intentions in young adults. In line with our hypothesis, overall public stigma around mental health and formal help-seeking intentions appeared to improve for those in the intervention group immediately following the intervention. These effects, however, were not sustained at follow-up. Contrary to our hypothesis, the contact-based intervention had a limited impact on improving self-stigma or informal help-seeking intentions across the whole sample. Although immediate improvements in self-stigma were not observed for either group postintervention, individuals who received the intervention demonstrated small improvements in self-stigma 1 week after the intervention. Finally, quantitative and qualitative results showed differing effectiveness of the intervention, with those experiencing high mental health distress responding less positively than those with low mental health distress, contrary to predictions.

Overall, these findings suggest that watching the brief videos can potentially lead to immediate reductions in public stigma and contemporaneous increases in the intentions to seek help from formal sources of help, such as a mental health professional, phone helpline, GP, or teacher. As these effects were not maintained at 1-week follow-up, the intervention’s impact on public stigma and help-seeking intentions appears to be short-lived. Conversely, a sleeper effect was observed for self-stigma, with improvements in self-stigma only emerging at follow-up. While previous reviews and meta-analyses have found contact effects to persist at short-term follow-up [7], others have not [8]. These findings may not be surprising given the intervention brevity and the small initial effect. Due to dropout, a lack of power at follow-up due to insufficient sample size may have also influenced the null findings relating to self-stigma and help-seeking from informal sources of help.

Recent meta-analyses of contact interventions yield small to moderate effects; however, the improvement in public stigma in this study was of small effect. It may be the case that the “dose” of the current intervention, at only approximately 8 minutes in duration, influenced the magnitude of the effect. Previously evaluated interventions generally range from 30 minutes to hours in duration [1,7-9]. Although the meta-analysis conducted by Morgan and colleagues [8] did not find that the duration of interventions influenced effectiveness, the review only examined studies targeting severe psychiatric stigma. One consideration in this study that may have contributed to the small effect of the brief digitized contact-based intervention on young adults is the isolated context in which the video was delivered. Typically, the lived experience stories are embedded within a wider batyr@uni program (60‐90 min in duration), which involves a live debrief discussion on key themes and actionable takeaways from the lived experience stories. While the brief videos used in this study include a prerecorded debrief, perhaps integrating a live facilitator to draw links between the story and discussions around key mental health themes may play a greater role in the intervention’s overall impact in the future. Evidence suggests that contact-based interventions are more effective when combined with psychoeducation components to improve mental health literacy [12], including approaches that explicitly target misconceptions regarding treatment-seeking [14], which may be more compelling for young people [14].

Shifting our focus to more nuanced exploratory findings within the subgroups, although both individuals with high and low mental health distress showed improvements in public stigma immediately following the intervention, the findings suggest that individuals with high mental health distress were less likely to want to seek help from informal sources of help, such as through a partner, friend, parent, relative, or family member, 1 week later. This finding is potentially concerning in the absence of an increase in formal help-seeking intentions in this subgroup, with no significant effect of intentions to seek help from formal sources such as a mental health professional, phone helpline, family doctor, GP, or teacher. It is these formal sources of help that have a higher likelihood, although far from guaranteed, to be evidence-based compared to help that may come from friends and family [35]. In contrast, the findings suggest that individuals with low mental health distress were more likely to use formal sources of help immediately after watching the brief video intervention; however, these effects were not sustained at follow-up. Further caution is warranted when interpreting the results, due to the exploratory and underpowered nature of the subgroup analyses or may reflect the complex interactions with prior mental health experiences. Additionally, the low reliability of the informal help-seeking subscale in the GHSQ suggests that these results should be interpreted with caution. Nonetheless, given that contact-based interventions are designed to increase help-seeking attitudes, particularly for individuals experiencing mental health difficulties who may need it the most, careful consideration in the design of brief digitized interventions is needed and tailoring the videos to target these vulnerable groups and encourage help-seeking would be beneficial.

Furthermore, the sleeper effect observed in improved self-stigma for all those who received the intervention was not evident in the subgroup analyses. No intervention effects at postintervention or follow-up were observed in self-stigma for either those with high or low mental health distress. This is not surprising given the small effect size observed in the whole sample (0.2). An insufficient sample size and subsequent lack of power in the subgroups may have contributed to the null findings for self-stigma. Regardless, these effects may indicate that the current intervention is not sufficiently powerful to shift young adults’ perception of their mental health. Future research should focus on replication with a larger sample and enhancing the intervention to allow for more meaningful and long-term shifts in mental health stigma.

Several key themes in the qualitative results were identified, showing differences between the subgroups on topics related to the helpfulness of the intervention and relatability to the speaker in the videos. For instance, individuals with high mental health distress tended to have more negative views about the helpfulness of the intervention and were less likely to be able to relate to the speaker’s experience in the videos. On the contrary, individuals with low mental health distress reported more positive responses to the intervention and found the videos educational and helpful. These qualitative findings provide valuable insights into young adults’ perceptions of the digitized contact-based intervention. While improvements in public stigma were observed in individuals with high mental health distress, they appeared to respond more negatively toward the contact-based intervention compared to those with low mental health distress. They reported that the intervention provided them with no additional information or learning, that they were familiar with the content conveyed, leaving little impact and negatively reinforced the stereotype that people can be cruel toward individuals with mental health difficulties who are perceived as different. This supports previous research indicating that contact-based interventions are less effective for individuals with prior mental health difficulties, which may be related to preexisting awareness and understanding [22]. A potential explanation may be that these individuals were less likely to see the value in seeking help from and disclosing their mental health to family members or friends following the intervention. These findings add to the growing literature indicating differences in perceptions between those with and without mental health difficulties [1,21,22].

Some participants in this study expressed their belief that the contact-based intervention would be helpful for others. Individuals with low mental health distress reported that they found the intervention helpful, educational, and insightful. A possible interpretation for this positive response may be that individuals with low mental health distress feel more connected and less alone after hearing about other people’s lived experiences of mental health difficulties. This is important to consider. If contact-based interventions are less effective for individuals with high mental health distress, future research could focus on alternative interventions or tailoring the content to focus less on education and awareness and more on targeting stigma and feelings of shame and embarrassment, which may be more appropriate to increase and improve help-seeking in young adults. This aligns with previous qualitative research, where individuals with a previous mental health concern were found to identify with themes of shame and embarrassment, compared to those without a previous mental health history [1].

Another factor to consider in designing these interventions pertains to the relatability of the speaker in the contact-based intervention. While the lived experience stories delivered within the batyr@uni program are represented by individuals from diverse backgrounds and experiences, these findings suggest that young adults’ relatability to the video speaker differs depending on their levels of mental health distress. Individuals with high mental health distress related less and responded less positively to the speaker. In contrast, participants with low mental health distress spoke more positively, often describing the speaker as highly relatable, genuine, and engaging. This discrepancy may contribute to differences in the effectiveness of the contact-based intervention between groups, suggesting different underlying mechanisms driving change in individuals. Perhaps individuals with high mental health distress experience unique challenges and find the more positive or uplifting aspects of the intervention to be somewhat incongruent with their current mental state. While individuals with low mental health distress may be able to connect more to the positive aspects of the speaker’s journey and view the speaker more favorably, and thus are more likely to seek help from formal sources of help as indicated in the quantitative subgroup analysis. Furthermore, the videos and the speaker delivering the intervention were not piloted for likability and perceived relatability prior to the intervention, which are factors shown to influence the effectiveness of antistigma interventions and may potentially account for group differences in perceived helpfulness, help-seeking intentions, and stigma among the low and high mental health distress groups. These are important aspects to consider when developing and evaluating the effectiveness of stigma-related interventions for young adults with mental health difficulties. Further research is needed to determine whether altering such factors may improve the efficacy of such interventions.

### Strengths, Limitations, and Future Directions

This study was the first to investigate whether a brief digitized contact-based intervention (one component of the batyr@uni program) was effective in reducing public stigma, self-stigma, and help-seeking intentions in a young adult university sample using a mixed methods study design. The quantitative and qualitative analyses provide a deeper insight into young adults’ perspectives of a brief digitized contact-based intervention targeting anxiety and depression. However, one limitation in this study is the way in which some constructs were measured. For instance, we did not measure actual help-seeking behavior, mental health difficulties, and the construct measurement of self-stigma was more aligned with anticipated self-stigma (ie, future-tense items may reduce sensitivity to change for participants without a current stigmatized identity), which may help explain stability in scores across time points. While help-seeking intentions predict behaviors [29], the relationship is not always strong [36]. Future research should prioritize behavioral measurement of actual help-seeking behavior to better evaluate intervention outcomes of such clinical significance.

Moreover, studies have found that factors such as openness to seeking help, increased mental health literacy, including recognition of disorder symptomology and treatment benefits, predicted help-seeking intentions within an undergraduate sample [12]. Further research is needed to determine whether additional components to contact alone, such as mental health literacy and openness to help-seeking, are needed to bridge the gap between stigma improvement and help-seeking attitudes [12].

In this study, approximately 1 in 5 participants dropped out prior to completing assessments at postintervention, and of those who did, over half did not complete the follow-up assessment. Although there were no differences at baseline or across conditions for dropout, it is difficult to determine whether any systematic factor influenced the dropout rate. As the study was completed online and anonymized, it was not possible to determine the reason for dropout. Such substantial dropout likely influenced the power to detect effects at that time point, possibly accounting for the absence of certain effects persisting over time.

A further limitation is that substantial baseline differences were identified between the intervention and control groups on outcome measures, despite randomization to condition after the completion of baseline measures. Given precision in the identical nature of the procedures between conditions and no differences in the way individuals progressed through the trial, it was concluded that these differences occurred by chance. Nevertheless, this impacted our analysis method and interpretation of results, which involved ANCOVA using baseline status on the outcome variable as a covariate to adjust for the baseline imbalance between groups.

The sample in this study limits the generalizability of findings to other similar groups of young adults. In particular, the small sample size of 12 participants in the qualitative study may not be representative of the perceptions and attitudes of the population from which they were drawn. Participants in this study were majority female undergraduate university students studying psychology, and thus, we might expect lower levels of mental health stigma and greater understanding or awareness when compared to the broader population. Relatedly, for the qualitative study, participants voluntarily expressed their interest in the study and willingness to share their perspectives. The participants involved may have been those more familiar with mental health stigma or those with strongly held opinions that they wished to discuss. Further, the brief contact intervention specifically targeted anxiety and depression in young adults; however, the nature and intensity of mental health stigma can differ across mental disorders, thus the findings may be limited to experiences with common mental disorders. Another limitation is due to the small sample of 6 participants in each group (high vs low mental health distress group). That said, the information elicited from these participants during the in-depth interviews was sufficiently rich to address the study objectives.

Despite these limitations, significant qualitative differences in this study were found among the sample of psychology students, perhaps indicating that greater differences may exist within the broader population. Future research should extend the evaluation of contact-based interventions beyond a university setting to young adults within the community and from different cultural and socioeconomic backgrounds. Social and cultural differences, as well as digital literacy factors, would likely appear more pronounced within diverse community settings and may weigh more heavily on digital intervention efficacy and moderators.

Given the benefits of a digitized and brief intervention in this study for those with low mental health distress, such as cost-effectiveness, accessibility, and convenience, more widespread dissemination of contact-based interventions appears possible. This has implications for when face-to-face dissemination is not feasible, such as government-mandated lockdowns, or for those living in rural and remote regions. Further research on the effectiveness of a digitized and brief intervention in varied populations would be useful.

### Conclusions

In conclusion, this study suggests that a brief contact-based video targeting anxiety and depression holds promise as an intervention that could be widely disseminated to reduce young adults’ mental health public stigma in the short term; however, further research evaluating its long-term impact is needed. The intervention had a limited effect on self-stigma. Although the intervention changed the way people viewed others with mental health difficulties, it did not change how individuals perceived their own mental health. In fact, there is some evidence that the intervention may lead young people with high mental distress to be less likely to speak to their friends and family. Together, these findings suggest the need for brief digitized contact-based interventions to be adapted to better cater to individuals with high mental health distress. Factors such as the perceived helpfulness of the intervention and relatability to the speaker in the videos were identified as key themes to consider when developing and disseminating contact-based interventions for young adults. Several study limitations may underlie null effects following the contact intervention for improvements in self-stigma and help-seeking intentions, and limit the extent to which findings may be extrapolated, indicating a need for further research and replication, particularly in more diverse populations. Further research is needed to determine whether tailored alternate strategies or adjunct components, such as individuals’ mental health literacy, prior experience with mental health difficulties, perceived likability of speakers delivering the videos, and behavioral measures of help-seeking, could improve outcomes, particularly in self-stigma and help-seeking intentions in those with high mental health distress.

## Supplementary material

10.2196/74391 Multimedia Appendix 1 Bespoke interview questions created for this study.

## Abbreviations

ANCOVA: analysis of covariance
DASS-21: Depression, Anxiety and Stress Scale - Short Form
GASS: Generalized Anxiety Stigma Scale
GHSQ: General Help-Seeking Questionnaire
GP: general practitioner
MHSS: Mental Health Stigma Scale
SSOMI: Self-Stigma of Mental Illness Scale
