# Supplementary material for: Effectiveness of a Brief Digitized Contact-Based Intervention in Improving Mental Health Stigma and Help-Seeking in Young Adults: Mixed Methods Study
Source: JMIR Hum Factors. 2026 Jun 10;13:e74391. doi: 10.2196/74391 (PMC13251860; doi:10.2196/74391)
Supplement: Multimedia Appendix 1 [file humanfactors-v13-e74391-s001.docx]

Participants in the intervention group were provided with an opportunity to express their interest in an interview to discuss their perspectives on the brief contact-based intervention. Participants were asked initial warm up questions relating to general mental health stigma, followed by questions relating to their previous relevant experiences, help-seeking attitudes, perspectives on the contact-based intervention, and thoughts about the speaker in the intervention. Below are the bespoke questions in relation to the intervention:

- Did you have any general thoughts about the study or the video?
- In terms of the video, did you have any thoughts about the speaker (the person who was sharing their mental health journey/experiences), or how did you find watching him/her share his/her story and talking about his/her experiences?
- Was there anything that the speaker shared that surprised you or made you think a bit differently or that you weren’t really aware of?
- Did you find yourself relating to anything the speaker said or to the speaker himself?
- How do you think other people would have perceived the video or respond to the video?
- Where there any aspects of the video that you liked or didn’t like?
- Generally speaking, what do you think could be done to help improve or reduce mental health stigma?
- Do you think a video like this could be helpful or useful for others?
